# Supplementary material for: Content-rich biological network constructed by mining PubMed abstracts
Source: BMC Bioinformatics. 2004 Oct 8;5:147. doi: 10.1186/1471-2105-5-147 (PMC528731; doi:10.1186/1471-2105-5-147)
Supplement: Additional File 2 — The original results of the above study (non-essential files are deleted to keep the file size under the limit set by BMC bioinformatics). [file 1471-2105-5-147-S2.bz2 › chilibotAdditionalFile2/dip10/288ID1836978E1118/html/CCNB1_CDC25A.html]

 


 **CCNB1** and **CDC25A** 
  
Found 11 abstracts in PubMed, retrieved 10.  
 

 What does Google say? 
 PDF only 
| .edu only 

---

**Interactive relationship** (e.g. stimulation, inhibition, etc)

**Inhibitory relationship**- Consistent with this, the levels of cyclin B  [ **CCNB1** ]  Cdc2 and cyclin E Cdk2 histone H1 kinase activity were both reduced by approximately 50% after phosphatase dead  **Cdc25A**  injection.  Ref: 10433828 Dev Biol, 1999
**Neutral relationship**- **Cdc25A**  binds and activates cyclin B  [ **CCNB1** ]  Cdk1, accelerates cell division when overexpressed, and its downregulation by RNA interference RNAi delays mitotic entry.  Ref: 12411508 EMBO J, 2002
- While  **Cdc25A**  Cys > Ser could interact with cyclin A Cdk2, cyclin B  [ **CCNB1** ]  Cdc2, and cyclin E Cdk2 strongly, Cdc25B mutant was only found to bind to cyclin A Cdk2 at significant levels.  Ref: 8617791 J Biol Chem, 1996

**Non-interactive relationship** (e.g. studied together, co-existance, homology, etc.)

- With respect to  **CDC25A** , cyclin D1, cyclin B1  [ **CCNB1** ] , and Ki 67, no statistically significant differences were found in their expressions between SR and RR tumors.  Ref: 11156245 Clin Cancer Res, 2000
- Their preradiation biopsy samples were immunohistochemically investigated for the expressions of cell cycle related molecules, including p53,  **CDC25A** , CDC25B, cyclin D1, cyclin B1  [ **CCNB1** ] , and Ki 67.  Ref: 11156245 Clin Cancer Res, 2000
- Furthermore, SA also reduced the expressions of  **Cdc25A**  and cyclin B  [ **CCNB1** ] , blocked cell cycle progression at G2 M phase, and induced apoptosis in SiHa cells.  Ref: 12054599 Biochem Biophys Res Commun, 2002
- Mitotic stabilization of  **Cdc25A**  reflects its phosphorylation on Ser17 and Ser115 by cyclin B  [ **CCNB1** ]  Cdk1, modifications required to uncouple  **Cdc25A**  from its ubiquitin proteasome mediated turnover.  Ref: 12411508 EMBO J, 2002
